
JOURNAL INFORMATION
==============================
NLM Title Abbreviation: ScientificWorldJournal
Journal ID: ScientificWorldJournal
NLM Journal ID: 101131163
Journal ID: TSWJ

The Scientific World Journal

ISSN: 2356-6140
EISSN: 1537-744X
Publisher: Wiley

ARTICLE INFORMATION
==============================
PMCID: PMC5956443
PMID: 15517096
DOI: 10.1100/tsw.2004.138
Article ID: 954967
Article version: 1
Subjects: Case Study

Incidental Discovery of Emphysematous Cystitis with Rapid Resolution in a Patient Presenting with Acute Pancreatitis

Al-Assiri M. *
Chan P.
Department of Urology, Royal Victoria Hospital, McGill University Health Centre, Montreal, Quebec, Canada
*M. Al-Assiri: manaalassiri@yahoo.comAcademic Editor: Anthony Atala

Publication date: 2004
Electronic publication date: 2004 Sep 30
Volume: 4
First page: 881
Last page: 884
Received 2004 Jul 26; Revised 2004 Aug 27; Accepted 2004 Aug 30
Copyright: Copyright © 2004 M. Al-Assiri and P. Chan.
Copyright year: 2004
License: This is an open access article distributed under the Creative Commons Attribution License, which permits unrestricted use, distribution, and reproduction in any medium, provided the original work is properly cited.
License URL: https://creativecommons.org/licenses/by/3.0/

Keywords: cystitis, bladder disease, emphysema, pancreatitis

==============================
Case Report
*TheScientificWorldJOURNAL* (2004) 4, 881–884
ISSN 1537-744X; DOI 10.1100/tsw.2004.138

### Incidental Discovery of Emphysematous Cystitis with Rapid Resolution in a Patient Presenting with Acute Pancreatitis

M. Al-Assiri* and P. Chan
*Department of Urology, Royal Victoria Hospital, McGill University Health Centre, Montreal, Quebec, Canada*

**E-mail:** manaalassiri@yahoo.com

*Received July 26, 2004; Revised August 27, 2004; Accepted August 30, 2004; Published September 30, 2004*

**KEYWORDS:** cystitis,bladder disease,emphysema,pancreatitis

**DOMAIN**: urology

### CASE HISTORY

A 58-year-old male presented with a 2-h history of epigastric pain, nausea, and vomiting after an episode of heavy alcohol consumption. He denied any irritative and obstructive urinary symptoms.

On physical examination:

1. Afebrile with stable vital signs.
2. Abdominal examination revealed epigastric tenderness with rebound.

Investigation:

1. Elevated lipase level (90 U/L), his serum biochemical profile was essentially normal.
2. A urinalysis was positive for red blood cells, negative for white blood cells and nitrites.

A clinical diagnosis of acute pancreatitis was made.

A computerized tomography of the abdomen and pelvis was consistent with a Grade A pancreatitis (Fig. 1). Incidentally, air was noted within the urinary bladder wall (Fig. 2). Of note, there was no recent urinary tract instrumentation.

The diagnosis was **emphysematous cystitis**.

The patient was treated with oral antibiotics with prompt clinical response. Three days later, a follow-up CT abdomen and pelvis showed significant resolution of the emphysema in urinary bladder (Fig. 3). No significant change was noted in appearance of the pancreas (Fig. 4).

This case shows that a life-threatening urological condition could present with a nonurological complaint.

*Corresponding author.
©2004 with author.

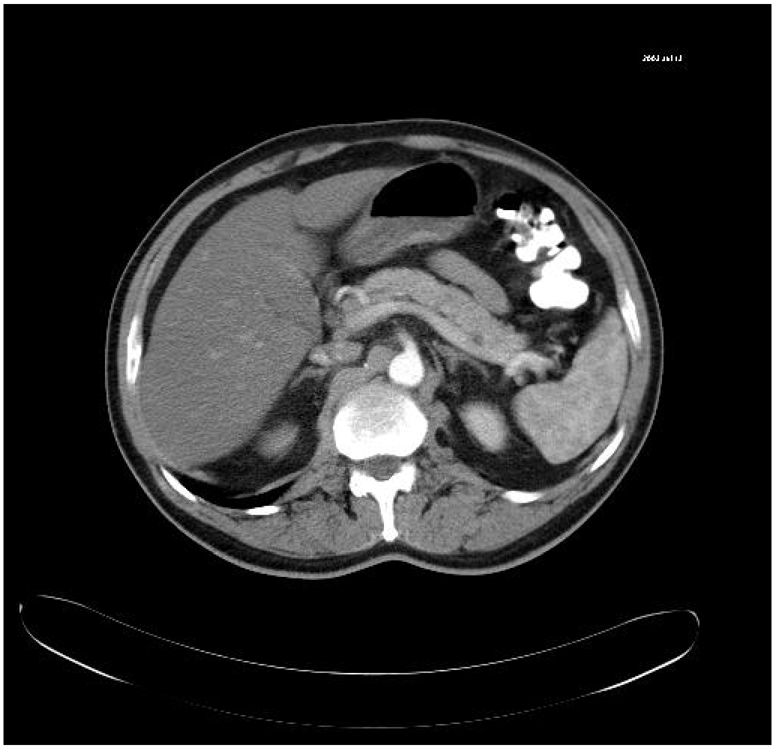

**FIGURE 1**. CT scan of the upper abdomen and pelvic demonstrating Grade A pancreatitis.

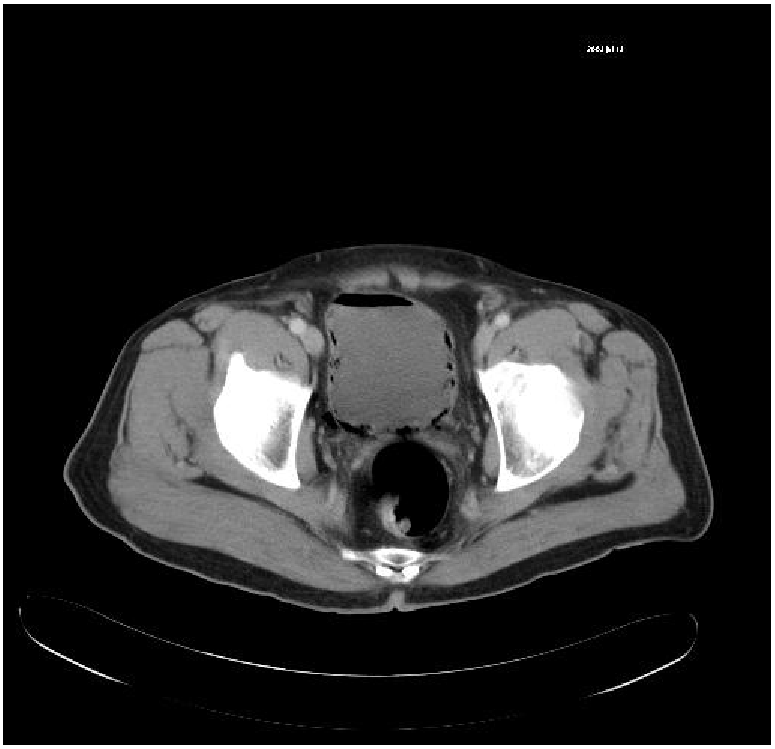

**FIGURE 2**. Intramural and intraluminal gas in the urinary bladder.

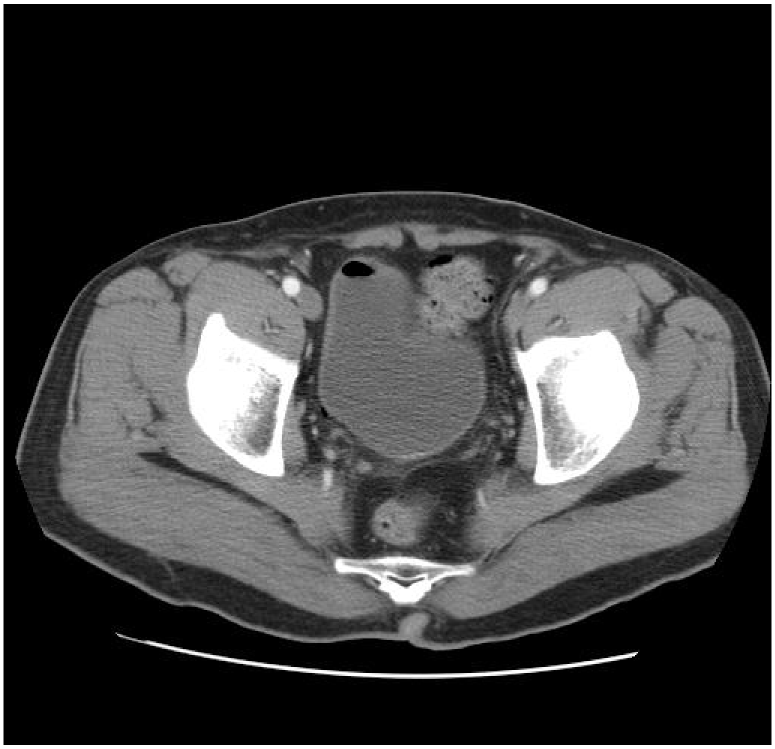

**FIGURE 3**. A follow-up CT obtained 3 days later demonstrated nearly a complete resolution of the intramural and intraluminal gas in the urinary bladder.

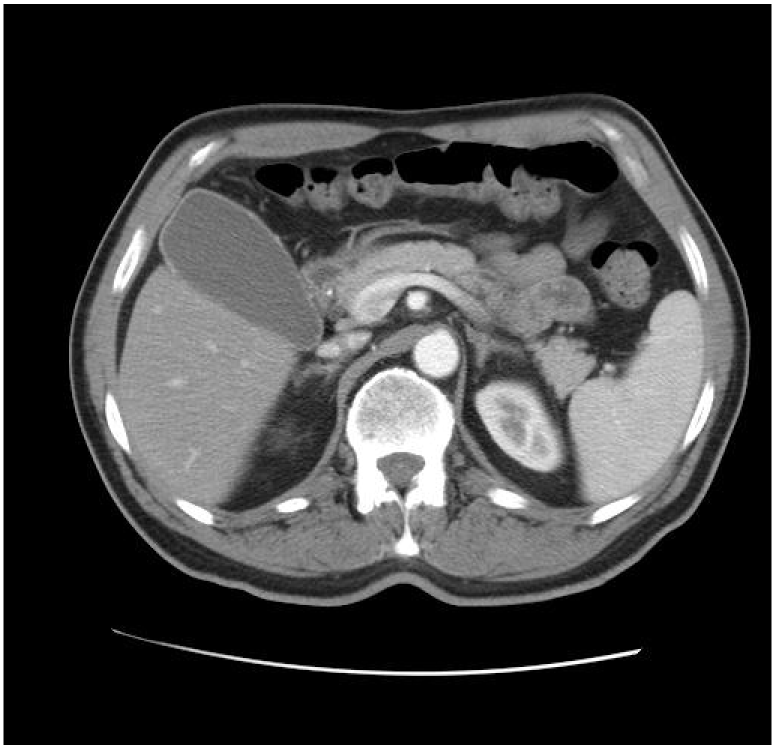

**FIGURE 4.** No significant change was observed in the appearance of the pancreas.

**This article should be referenced as follows:**

Al-Assiri, M. and Chan, P. (2004) Incidental discovery of emphysematous cystitis with rapid resolution in a patient presenting with acute pancreatitis. *TheScientificWorldJOURNAL* **4**, 881–884.

**Handling Editor:**

Anthony Atala, Principal Editor for *Urology* — a domain of *TheScientificWorldJOURNAL*.
